# Ontario Veterinary Medical Association

**Published:** 1898-01

**Authors:** 


Secretary.

### THE MISSOURI VALLEY VETERINARY MEDICAL ASSOCIATION.

### ONTARIO VETERINARY MEDICAL ASSOCIATION.

THE annual meeting was held on December 24, 1897, at the Veterinary College. Major Lloyd, of Newmarket, was in the chair, and there was a fair attendance of members from the different parts of the Province. At the morning session the usual routine business was disposed of, and Mr. C. L. Smith, of Brantford, was elected a member.

When the afternoon meeting opened, Mr. W. J. Wilson drew the attention of the Association to a requisition by the Canadian Humane Society to Parliament, asking that the docking of horses' tails be prohibited except by a veterinary surgeon or by a student acting under the immediate orders of a veterinary surgeon. The Humane Society set forth that it was not possible for them to hope to obtain legislation at present prohibiting this practice entirely, but they desired to have a law passed which would make the pain of the animals as little as possible. The meeting indorsed the intention of the petition.

Attention was drawn by Mr. C. Elliott, of St. Catharine's, to the appointment of Lieutenant-Colonel McCrae to go through the Province and instruct farmers in the disease of tuberculosis in stock and the use of tuberculin. Why, he asked, did not the Government appoint Prof. Reed, of Guelph Agricultural College, who, the speaker said, had plenty of time to discharge such duties? The reason was that this was a first-class opportunity to give Colonel McCrae a job. The speaker said that he would not, and he did not think the profession would, indorse such an appointment, especially when there was a veterinary college with a number of thoroughly competent professors.

Mr. J. E. Blackhall, of Clinton, was very emphatic in his condemnation of the appointment of Colonel McCrae to this position. He considered that Hon. John Dryden had by the appointment "thrown down" the veterinary profession. He considered that unless a bill could be introduced and engineered through the House by some private member cold water would still continue to be poured on the back of the profession.

Mr. W. Gibb, of St. Mary's, said that Hon. Mr. Dryden had broken aloof from giving countenance to the veterinary profession by his action, and he for one was not disposed to submit tamely to it.

Mr. W. J. Wilson, of London, was inclined to think that the putting of tuberculin into the hands of the farmers in the way it would be if the proposal of the Government were carried out would not be in the interest of the live-stock trade of the country. The tuberculin could be used so as to deceive the Government inspectors and eventually destroy reliance in the stock of the Province.

After some further discussion a committee, composed of Messrs. Martin, Sisson, Cowan, and Elliott, was appointed to draft a resolution in regard to this matter.

The election of officers was then proceeded with, and resulted as follows: President, S. Sisson, Toronto; First Vice-President, W. J. Wilson, London; Second Vice-President, J. E. Blackhall, Clinton; Secretary-Treasurer, C. H. Sweetapple, Toronto; Directors, W. Steele, Stratford; G. W. Coulter, Weston; H. S. Wende, Tonawanda; W. Cowan, Galt; Orr Graham, Port Perry; W. Gibbs, St. Mary's; J. Wagner, Tavistock; E. Daly, Sutton. Delegates to Western Show Association, J. H. Wilson, London; J. D. O'Neil, London. Delegates to the Industrial Fair at Toronto, Dr. Andrew Smith and President Sisson. Auditors, J. D. O'Neil and C. Elliott, of St. Catharine's.

The resolution of the special committee regarding the appointment of Colonel McCrae was then read and adopted unanimously. It was as follows: "That the tuberculin-test requires to be safeguarded by certain precautions not possible to those without professional experience; that it is not in the interest of farmers of the Province to have any person authorized to instruct them in the use of tuberculin as a test for tuberculosis, as it is liable to create an unnecessary alarm concerning the health of the cattle of Ontario; that a continuation of the present methods will bring the tuberculin-test into disrepute, for the following reasons, namely, that animals destined for shipment to the United States may be injected with tuberculin by the owners or dealers, and subsequent tests by the Dominion veterinary inspector rendered both useless and deceptive, paving the way for fraud, and consequently injuring the live-stock trade of the country. If such appointment as proposed above were necessary, we disapprove of the action of the Minister of Agriculture in appointing Lieutenant-Colonel McCrae to that position instead of a duly qualified veterinary surgeon.

The report of the Secretary-Treasurer showed a favorable condition of the finances.

